# On the Connection Coefficients of the Chebyshev-Boubaker Polynomials

**DOI:** 10.1155/2013/657806

**Published:** 2013-08-04

**Authors:** Paul Barry

**Affiliations:** School of Science, Waterford Institute of Technology, Waterford, Ireland

## Abstract

The Chebyshev-Boubaker polynomials are the orthogonal polynomials whose coefficient arrays are defined by ordinary Riordan arrays. Examples include the Chebyshev polynomials of the second kind and the Boubaker polynomials. We study the connection coefficients of this class of orthogonal polynomials, indicating how Riordan array techniques can lead to closed-form expressions for these connection coefficients as well as recurrence relations that define them.

## 1. Introduction

In this paper, we will show that the usual recurrence relations that define elements of Riordan arrays can be used to give a set of recurrence relations for the connection coefficients of families of orthogonal polynomials defined by Riordan arrays. It will also be seen that we can give closed-form expressions for the connection coefficients themselves, at least so far, as it is easy to give closed-form expressions for the Riordan arrays concerned. Thus, in the special case of orthogonal polynomials that are defined by Riordan arrays, we can achieve two of the main goals involved in the study of connection coefficients for orthogonal polynomials [1–7].

If *P*_*n*_(*x*) and *Q*_*n*_(*x*) are two families of orthogonal polynomials, the connection coefficients *a*_*n*,*k*_ of *P*_*n*_ in terms of *Q*_*n*_, defined by

(1) $$\begin{array}{c} P_{n}=\sum_{k=0}^{n}a_{n,k}Q_{k}, \end{array}$$

are often of interest. In terms of matrices, we have

(2) $$\begin{array}{c} P=AQ, \end{array}$$

where **P** is the coefficient array of the polynomials *P*_*n*_(*x*), with elements *p*_*n*,*k*_, and **Q** is the coefficient array of the polynomials *Q*_*n*_(*x*), with elements *q*_*n*,*k*_. Thus, we have

(3) $$\begin{array}{c} P_{n}\left(x\right)=\sum_{k=0}^{n}p_{n,k}x^{k},\;\;Q_{n}\left(x\right)=\sum_{k=0}^{n}q_{n,k}x^{k}. \end{array}$$

The matrices **P** and **Q** are lower-triangular matrices. Clearly, the matrix **A** of connection coefficients is then given by

(4) $$\begin{array}{c} A=PQ^{-1}. \end{array}$$

We immediately note that

(5) $$\begin{array}{c} A^{-1}=QP^{-1}, \end{array}$$

so that the elements of **A** ^−1^ are the connection coefficients of *Q*_*n*_ in terms of *P*_*n*_.

The matrix **A** is lower triangular since both **P** and **Q** are.

We will define a family of orthogonal polynomials *P*_*n*_(*x*) to be of *Chebyshev-Boubaker* type, if the coefficient array **P** = (*p*_*n*,*k*_) is a Riordan array of the type

(6) $$\begin{array}{c} \left(\frac{1+ux+vx^{2}}{1+ax+bx^{2}},\frac{\lambda x}{1+ax+bx^{2}}\right). \end{array}$$

This means that

(7) $$\begin{array}{c} p_{n,k}=\left[x^{n}\right]\frac{1+ux+vx^{2}}{1+ax+bx^{2}}{\left(\frac{\lambda x}{1+ax+bx^{2}}\right)}^{k}. \end{array}$$

In the case that *λ* = 1, the polynomials are monic. An array of the type

(8) $$\begin{array}{c} \left(\frac{1+ux+vx^{2}}{1+ax+bx^{2}},\frac{\lambda x}{1+ax+bx^{2}}\right) \end{array}$$

will be called a *Chebyshev-Boubaker array* (with parameters (*a*, *b*, *u*, *v*, *λ*)). This is a generalization of the notion of a Chebyshev-Boubaker array considered in [8]. The Boubaker polynomials [8] correspond to the parameters (0,1, 0,3, 1).

**Example 1**

The Chebyshev polynomials of the second kind *U*_*n*_(*x*) are defined by

(9) $$\begin{array}{c} U_{n}\left(x\right)=\sum_{k=0}^{\left\lfloor n/2\right\rfloor }{\left(-1\right)}^{k}\left(\begin{array}{c} n-k\\ k \end{array}\right){\left(2x\right)}^{n-2k} \end{array}$$

and have coefficient array **U** given by A053117

(10) $$\begin{array}{c} U=\left(\frac{1}{1+x^{2}},\frac{2x}{1+x^{2}}\right). \end{array}$$

**Example 2**

We let *S*_*n*_(*x*) = *U*_*n*_(*x*/2) be the monic Chebyshev polynomials of the second kind. The coefficient array **S** of these polynomials is given by A049310

(11) $$\begin{array}{c} S=\left(\frac{1}{1+x^{2}},\frac{x}{1+x^{2}}\right). \end{array}$$

This is the case *u* = *v* = *a* = 0, *b* = 1, and *λ* = 2.

The moments *m*_*n*_ of this family of orthogonal polynomials are the aerated Catalan numbers that begin with

(12) $$\begin{array}{c} 1,0,1,0,2,0,5,0,14,0,42,\ldots . \end{array}$$

Their moment representation is given by

(13) $$\begin{array}{c} m_{n}=\frac{1}{2\pi }\int_{-2}^{2}x^{n}\sqrt{4-x^{2}}\;dx. \end{array}$$

Very often, orthogonal polynomials are specified in terms of the three-term recurrence that they satisfy. In the monic case, this will take the form

(14) $$\begin{array}{c} P_{n}\left(x\right)=\left(x-\alpha_{n}\right)P_{n-1}\left(x\right)-\beta_{n}P_{n-2}\left(x\right), \end{array}$$

with suitable initial conditions. For instance, we have

(15) Sn(x)=(x−2)Sn−1(x)−Sn−2(x),S0(x)=1,    S1(x)=x.

The recurrence coefficients *α*_*n*_ and *β*_*n*_ for the family *P*_*n*_ of orthogonal polynomials are the entries in the production matrix (or Stieltjes matrix [9]) of the matrix **P** ^−1^. This production matrix is equal to $P\bar{P^{-1}}$, where the notation $\bar{M}$ denotes the matrix **M** with its first row removed. For instance, the production matrix of **U** ^−1^ = (1/(1+*x* ^2^),2*x*/(1+*x* ^2^))^−1^ is given by

(16) $$\begin{array}{c} \left(\begin{array}{ccccccc} 0 & \frac{1}{2} & 0 & 0 & 0 & 0 & \cdots \\ \frac{1}{2} & 0 & \frac{1}{2} & 0 & 0 & 0 & \cdots \\ 0 & \frac{1}{2} & 0 & \frac{1}{2} & 0 & 0 & \cdots \\ 0 & 0 & \frac{1}{2} & 0 & \frac{1}{2} & 0 & \cdots \\ 0 & 0 & 0 & \frac{1}{2} & 0 & \frac{1}{2} & \cdots \\ \vdots & \vdots & \vdots & \vdots & \vdots & \vdots & \ddots \end{array}\right), \end{array}$$

corresponding to the three-term recurrence

(17) $$\begin{array}{c} \frac{1}{2}U_{n}\left(x\right)=\left(x-0\right)U_{n-1}\left(x\right)-\frac{1}{2}U_{n-2}\left(x\right), \end{array}$$

or

(18) $$\begin{array}{c} U_{n}\left(x\right)=2xU_{n-1}\left(x\right)-U_{n-2}\left(x\right). \end{array}$$

Similarly, the production matrix of **S** ^−1^ = (1/(1+*x* ^2^), *x*/(1+*x* ^2^))^−1^ is given by

(19) $$\begin{array}{c} \left(\begin{array}{ccccccc} 0 & 1 & 0 & 0 & 0 & 0 & \cdots \\ 1 & 0 & 1 & 0 & 0 & 0 & \cdots \\ 0 & 1 & 0 & 1 & 0 & 0 & \cdots \\ 0 & 0 & 1 & 0 & 1 & 0 & \cdots \\ 0 & 0 & 0 & 1 & 0 & 1 & \cdots \\ \vdots & \vdots & \vdots & \vdots & \vdots & \vdots & \ddots \end{array}\right), \end{array}$$

corresponding to the three-term recurrence

(20) $$\begin{array}{c} S_{n}\left(x\right)=xS_{n-1}\left(x\right)-S_{n-2}\left(x\right). \end{array}$$

More generally, the production matrix of **S**_*b*_^−1^ = (1/(1+*bx* ^2^),*x*/(1+*bx* ^2^))^−1^ is given by

(21) $$\begin{array}{c} \left(\begin{array}{ccccccc} 0 & 1 & 0 & 0 & 0 & 0 & \cdots \\ b & 0 & 1 & 0 & 0 & 0 & \cdots \\ 0 & b & 0 & 1 & 0 & 0 & \cdots \\ 0 & 0 & b & 0 & 1 & 0 & \cdots \\ 0 & 0 & 0 & b & 0 & 1 & \cdots \\ \vdots & \vdots & \vdots & \vdots & \vdots & \vdots & \ddots \end{array}\right), \end{array}$$

corresponding to the three-term recurrence

(22) Sn(b)(x)=xSn−1(b)(x)−bSn−2(b)(x),S0(b)=1,  S1(b)(x)=x,

where

(23) $$\begin{array}{c} S_{n}^{\left(b\right)}\left(x\right)=\sum_{k=0}^{\left\lfloor n/2\right\rfloor }\left(\begin{array}{c} n-k\\ k \end{array}\right){\left(-b\right)}^{k}x^{n-2k}. \end{array}$$

**Example 3**

The Boubaker polynomials [8, 10–15] have coefficient array

(24) $$\begin{array}{c} \left(\frac{1+3x^{2}}{1+x^{2}},\frac{x}{1+x^{2}}\right). \end{array}$$

They can be expressed as

(25) $$\begin{array}{c} P_{n}\left(x\right)=\sum_{k=0}^{\left\lfloor n/2\right\rfloor }\left(\begin{array}{c} n-k\\ k \end{array}\right)\frac{n-4k}{n-k}{\left(-1\right)}^{k}x^{n-2k}. \end{array}$$

We have

(26) (1+3x21+x2,x1+x2)−1=(1+x2c(x2)21+3x2c(x2)2,xc(x2))=(11+2x2c(x2),xc(x2)),

where

(27) $$\begin{array}{c} c\left(x\right)=\frac{1-\sqrt{1-4x}}{2x} \end{array}$$

is the generating function of the Catalan numbers

(28) $$\begin{array}{c} C_{n}=\frac{1}{n+1}\left(\begin{array}{c} 2n\\ n \end{array}\right). \end{array}$$

Hence, the moments *m*_*n*_ of this family of orthogonal polynomials have g.f. 1/(1 + 2*x* ^2^c(*x* ^2^)). The sequence *m*_*n*_ begins with

(29) $$\begin{array}{c} 1,0,-2,0,2,0,-4,0,2,0,-12,0,-12,0,-72,\ldots \end{array}$$

and has the moment representation

(30) $$\begin{array}{c} m_{n}=-\frac{1}{\pi }\int_{-2}^{2}x^{n}\frac{\sqrt{4-x^{2}}}{4+3x^{2}}\;dx+\frac{2}{3}{\left(-\frac{2}{\sqrt{3}}i\right)}^{n}+\frac{2}{3}{\left(\frac{2}{\sqrt{3}}i\right)}^{n}. \end{array}$$

We have

(31) m2n=∑k=0nk+0n+kn+0nk(2n−k−1n−k)(−2)k=∑k=0n2k+1n+k+1(2nn−k)(−3)k.

The sequence *m*_2*n*_ is A126984. We note that the Hankel transforms of *m*_*n*_ and *m*_2*n*_ are both equal to (−2)^*n*^. The production matrix of ((1 + 3*x* ^2^)/(1+*x* ^2^),*x*/(1+*x* ^2^))^−1^ is given by

(32) $$\begin{array}{c} \left(\begin{array}{ccccccc} 0 & 1 & 0 & 0 & 0 & 0 & \cdots \\ -2 & 0 & 1 & 0 & 0 & 0 & \cdots \\ 0 & 1 & 0 & 1 & 0 & 0 & \cdots \\ 0 & 0 & 1 & 0 & 1 & 0 & \cdots \\ 0 & 0 & 0 & 1 & 0 & 1 & \cdots \\ \vdots & \vdots & \vdots & \vdots & \vdots & \vdots & \ddots \end{array}\right). \end{array}$$

In this paper, we will give a closed-form expression for the connection coefficients of the Chebyshev-Boubaker polynomials *P*_*n*_(*x*) with coefficient array

(33) $$\begin{array}{c} P=\left(\frac{1+ux+vx^{2}}{1+ax+bx^{2}},\frac{x}{1+ax+bx^{2}}\right) \end{array}$$

in terms of the Chebyshev-Boubaker polynomials *Q*_*n*_(*x*) with coefficient array

(34) $$\begin{array}{c} Q=\left(\frac{1+sx+tx^{2}}{1+cx+dx^{2}},\frac{x}{1+cx+dx^{2}}\right). \end{array}$$

We note that the polynomials *P*_*n*_(*x*) satisfy the recurrence

(35) $$\begin{array}{c} P_{n}\left(x\right)=\left(x-a\right)P_{n-1}\left(x\right)-bP_{n-2}\left(x\right),\;n\geq 3, \end{array}$$

with

(36) P0(x)=1,  P1(x)=x+u−a,P2(x)=x2+(u−2a)x−ua+a2+v−b,

with a similar expression for *Q*_*n*_(*x*).

In the next section, we recall notational elements that we will use in the sequel, along with a brief introduction to Riordan arrays.

## 2. Preliminaries on Integer Sequences and Riordan Arrays

In this section, we define terms used later to discuss integer sequences, Riordan arrays, production matrices, orthogonal polynomials, and Hankel transforms. Readers familiar with these areas and the links between them may skip this section.

For an integer sequence *a*_*n*_, that is, an element of *ℤ* ^*ℕ*^, the power series *f*(*x*) = ∑_*n*=0_^*∞*^ *a*_*n*_ *x* ^*n*^ is called the *ordinary generating function* or g.f. of the sequence. *a*_*n*_ is thus the coefficient of *x* ^*n*^ in this series. We denote this by *a*_*n*_ = [*x* ^*n*^]*f*(*x*). For instance, *F*_*n*_ = [*x* ^*n*^](*x*/(1 − *x* − *x* ^2^)) is the *n*th Fibonacci number A000045, while $C_{n}=[x^{n}](\left(1-\sqrt{1-4x}\right)/2x)$ is the *n*th Catalan number A000108. We use the notation 0^*n*^ = [*x* ^*n*^]1 for the sequence 1,0, 0,0,…,  A000007. Thus, $0^{n}=[n=0]=\delta_{n,0}=\left(\begin{array}{c} 0\\ n \end{array}\right)$. Here, we have used the Iverson bracket notation [16], defined by [*𝒫*] = 1 if the proposition *𝒫* is true and [*𝒫*] = 0 if *𝒫* is false.

For a power series *f*(*x*) = ∑_*n*=0_^*∞*^ *a*_*n*_ *x* ^*n*^ with *f*(0) = 0, we define the reversion or compositional inverse of *f* to be the power series $\bar{f}(x)$ such that $f(\bar{f}(x))=x$.

For a lower triangular matrix (*a*_*n*,*k*_)_*n*,*k*≥0_, the row sums give the sequence with general term ∑_*k*=0_^*n*^ *a*_*n*,*k*_. For a sequence *a*_*n*_, the sequence of determinants |*a*_*i*+*j*_|_0≤*i*,*j*≤*n*_ is called the Hankel transform of *a*_*n*_ [17–27]. 

The *Riordan group* [28, 29] is a set of infinite lower-triangular integer matrices, where each matrix is defined by a pair of generating functions *g*(*x*) = 1 + *g*_1_ *x* + *g*_2_ *x* ^2^ + ⋯ and *f*(*x*) = *f*_1_ *x* + *f*_2_ *x* ^2^ + ⋯ where *f*_1_ ≠ 0 [29]. We often require in addition that *f*_1_ = 1. The associated matrix is the matrix whose *i*th column is generated by *g*(*x*)*f*(*x*)^*i*^ (the first column being indexed by 0). The matrix corresponding to the pair *g*, *f* is denoted by (*g*, *f*) or *ℛ*(*g*, *f*). The group law is then given by

(37) $$\begin{array}{c} \left(g,f\right)\cdot \left(h,l\right)=\left(g,f\right)\left(h,l\right)=\left(g\left(h\circ f\right),l\circ f\right). \end{array}$$

The identity for this law is *I* = (1, *x*), and the inverse of (*g*, *f*) is

(38) $$\begin{array}{c} {\left(g,f\right)}^{-1}=\left(\frac{1}{g\circ \bar{f}}\;,\bar{f}\right), \end{array}$$

where $\bar{f}$ is the compositional inverse of *f*.

If **M** is the matrix (*g*, *f*) and **a** = (*a*_0_, *a*_1_,…)^*T*^ is an integer sequence (expressed as an infinite column vector) with ordinary generating function *𝒜*(*x*), then the sequence **M** **a** has ordinary generating function *g*(*x*)*𝒜*(*f*(*x*)). The (infinite) matrix (*g*, *f*) can thus be considered to act on the ring of integer sequences *ℤ* ^*ℕ*^ by multiplication, where a sequence is regarded as a (infinite) column vector. We can extend this action to the ring of power series *ℤ*[[*x*]] by

(39) $$\begin{array}{c} \left(g,f\right):𝒜\left(x\right)\mapsto \left(g,f\right)\cdot 𝒜\left(x\right)=g\left(x\right)𝒜\left(f\left(x\right)\right). \end{array}$$

**Example 4**

The so-called *binomial matrix B* is the element (1/(1 − *x*), *x*/(1 − *x*)) of the Riordan group. It has general element $\left(\begin{array}{c} n\\ k \end{array}\right)$ and hence as an array coincides with Pascal's triangle. More generally, *B* ^*m*^ is the element (1/(1 − *mx*), *x*/(1 − *mx*)) of the Riordan group, with general term $\left(\begin{array}{c} n\\ k \end{array}\right)m^{n-k}$. It is easy to show that the inverse *B* ^−*m*^ of *B* ^*m*^ is given by (1/(1 + *mx*), *x*/(1 + *mx*)).

The row sums of the matrix (*g*, *f*) have generating function

(40) $$\begin{array}{c} \left(g,f\right)\cdot \frac{1}{1-x}=\frac{g\left(x\right)}{1-f\left(x\right)}. \end{array}$$

**Example 5**

The inverse of the Riordan array (1/(1 + *x*), *x*/(1 + *x*)^2^) is the Riordan array (*c*(*x*), *xc*(*x*)^2^). This follows since the solution of the equation

(41) $$\begin{array}{c} \frac{z}{{\left(1+z\right)}^{2}}=x \end{array}$$

is given by

(42) $$\begin{array}{c} z=xc{\left(x\right)}^{2}. \end{array}$$

We then have

(43) $$\begin{array}{c} 1+xc{\left(x\right)}^{2}=c\left(x\right). \end{array}$$

Many interesting examples of sequences and Riordan arrays can be found in Neil Sloane's On-Line Encyclopedia of Integer Sequences (OEIS), [30, 31]. Sequences are frequently referred to by their OEIS number. For instance, the binomial matrix *B* (“Pascal's triangle”) is A007318.

For an invertible matrix *M*, its production matrix (also called its Stieltjes matrix) [32, 33] is the matrix

(44) $$\begin{array}{c} {𝒮}_{M}=M^{-1}\bar{M}, \end{array}$$

where $\bar{M}$ is the matrix *M* with its first row removed. A Riordan array *M* is the inverse of the coefficient array of a family of orthogonal polynomials [34–36] if and only if *𝒮*_*M*_ is tridiagonal [8, 17]. Necessarily, the Jacobi coefficients (i.e., the coefficients of the three-term recurrence [34] that defines the polynomials) of these orthogonal polynomials are then constant.

**Example 6**

The production matrix of (*c*(*x*), *xc*(*x*)^2^) is given by

(45) $$\begin{array}{c} \left(\begin{array}{ccccccc} 1 & 1 & 0 & 0 & 0 & 0 & \cdots \\ 1 & 2 & 1 & 0 & 0 & 0 & \cdots \\ 0 & 1 & 2 & 1 & 0 & 0 & \cdots \\ 0 & 0 & 1 & 2 & 1 & 0 & \cdots \\ 0 & 0 & 0 & 1 & 2 & 1 & \cdots \\ 0 & 0 & 0 & 0 & 1 & 2 & \cdots \\ \vdots & \vdots & \vdots & \vdots & \vdots & \vdots & \ddots \end{array}\right). \end{array}$$

An important feature of Riordan arrays is that they have a number of sequence characterizations [37, 38]. The simplest of these is as follows.

**Proposition 7 (see [38], Theorems 2.1, and 2.2)**

Let *D* = [*d*_*n*,*k*_] be an infinite triangular matrix. Then, *D* is a Riordan array if and only if there exist two sequences *A* = [*α*_0_, *α*_1_, *α*_2_,…] and *Z* = [*z*_0_, *z*_1_, *z*_2_,…] with *α*_0_ ≠ 0, *z*_0_ ≠ 0 such that

- *d*_*n*+1,*k*+1_ = ∑_*j*=0_^*∞*^ *α*_*j*_ *d*_*n*,*k*+*j*_, (*k*, *n* = 0,1,…);
- *d*_*n*+1,0_ = ∑_*j*=0_^*∞*^ *z*_*j*_ *d*_*n*,*j*_, (*n* = 0,1,…).

The coefficients *α*_0_, *α*_1_, *α*_2_,… and *z*_0_, *z*_1_, *z*_2_,… are called the *A*-sequence and the *Z*-sequence of the Riordan array *M* = (*g*(*x*), *f*(*x*)), respectively. Letting *A*(*x*) be the generating function of the *A*-sequence and *Z*(*x*) the generating function of the *Z*-sequence, we have

(46) $$\begin{array}{c} A\left(x\right)=\frac{x}{\bar{f}\left(x\right)},\;\;Z\left(x\right)=\frac{1}{\bar{f}\left(x\right)}\left(1-\frac{d_{0,0}}{g\left(\bar{f}\left(x\right)\right)}\right). \end{array}$$

The first column of *𝒮*_*M*_ is then generated by *Z*(*x*), while the *k*th column is generated by *x* ^*k*−1^ *A*(*x*) (taking the first column to be indexed by 0).

**Proposition 8**

The inverse of the Chebyshev-Boubaker array

(47) $$\begin{array}{c} P={\left(\frac{1+ux+vx^{2}}{1+ax+bx^{2}},\frac{1}{1+ax+bx^{2}}\right)}^{-1} \end{array}$$

has production matrix which begins with

(48) $$\begin{array}{c} \left(\begin{array}{ccccccc} a-u & 1 & 0 & 0 & 0 & 0 & \cdots \\ b-v & a & 1 & 0 & 0 & 0 & \cdots \\ 0 & b & a & 1 & 0 & 0 & \cdots \\ 0 & 0 & b & a & 1 & 0 & \cdots \\ 0 & 0 & 0 & b & a & 1 & \cdots \\ 0 & 0 & 0 & 0 & b & a & \cdots \\ \vdots & \vdots & \vdots & \vdots & \vdots & \vdots & \ddots \end{array}\right). \end{array}$$

**Proof**

We let **M** = (*g*(*x*), *f*(*x*)) = **P** ^−1^. Then,

(49) $$\begin{array}{c} A\left(x\right)=\frac{x}{\bar{f}\left(x\right)}=\frac{x}{x/\left(1+ax+bx^{2}\right)}=1+ax+bx^{2}. \end{array}$$

We also have

(50) Z(x)=1+ax+bx2x(1−1+ux+vx21+ax+bx2)=(a−u)+(b−v)x,

which proves the assertion.

## 3. Chebyshev-Boubaker Arrays

We can characterize the Chebyshev-Boubaker polynomials *P*_*n*_(*x*) with coefficient array **P** = ((1 + *ux* + *vx* ^2^)/(1 + *ax* + *bx* ^2^), *λx*/(1 + *ax* + *bx* ^2^)) as follows.

**Proposition 9 (see [8])**

The Chebyshev-Boubaker coefficient array ((1 + *ux* + *vx* ^2^)/(1 + *ax* + *bx* ^2^),*λx*/(1 + *ax* + *bx* ^2^)) is the coefficient array of the generalized Chebyshev polynomials *P*_*n*_(*x*) of the second kind given by

(51) Pn(x)=(b)nUn(λx−a2b)+u(b)n−1Un−1(λx−a2b) +v(b)n−2Un−2(λx−a2b).

The following variant is also true. The array ((1 + *ux* + *vx* ^2^)/(1 + *λ* *ax* + *bx* ^2^), *λx*/(1 + *λ* *ax* + *bx* ^2^)) is the coefficient array of the generalized Chebyshev polynomials of the second kind *P*_*n*_^(*λ*)^(*x*) given by

(52) Pn(λ)(x)=(b)nUn(λ(x−a)2b)+u(b)n−1Un−1 ×(λ(x−a)2b)+v(b)n−2Un−2(λ(x−a)2b).

The following factorization of the Chebyshev-Boubaker array will be useful when it comes to giving a closed-form expression for the connection coefficients.

**Proposition 10**

(The canonical factorization of Chebyshev-Boubaker arrays). Given the Chebyshev-Boubaker array

(53) $$\begin{array}{c} P=\left(\frac{1+ux+vx^{2}}{1+a\lambda x+bx^{2}},\frac{\lambda x}{1+a\lambda x+bx^{2}}\right), \end{array}$$

one has

(54) P=(1+ux+vx2,x)·(11+bx2,λx1+bx2) ·(11+ax,x1+ax).

**Proof**

The proof is a straight-forward application of the multiplication rule for Riordan arrays. For instance, we have

(55) (11+bx2,λx1+bx2)·(11+ax,x1+ax)  =(11+bx211+aλx/(1+bx2),    λx/(1+bx2)1+aλx/(1+bx2))  =(11+aλx+bx2,λx1+aλx+bx2).

For the monic case *λ* = 1, we have

(56) (1+ux+vx21+ax+bx2,x1+ax+bx2)  =(1+ux+vx2,x)·(11+bx2,x1+bx2)   ·(11+ax,x1+ax).

**Corollary 11**

If

(57) $$\begin{array}{c} P=\left(\frac{1+ux+vx^{2}}{1+a\lambda x+bx^{2}},\frac{\lambda x}{1+a\lambda x+bx^{2}}\right), \end{array}$$

then

(58) P−1=(11−ax,x1−ax)·(c(bx2),1λxc(bx2)) ·(11+ax+bx2,x).

In the monic case *λ* = 1, we then have

(59) (1+ux+vx21+ax+bx2,x1+ax+bx2)−1  =(11−ax,x1−ax)·(c(bx2),xc(bx2))   ·(11+ax+bx2,x).

We can characterize the Jacobi coefficients of *P*_*n*_(*x*) in terms of those of *Q*_*n*_(*x*) as follows. We let ${𝒮}_{P}=P\bar{P^{-1}}$ be the production matrix of **P** ^−1^, and we let ${𝒮}_{Q}=Q\bar{Q^{-1}}$ be the production matrix of **Q** ^−1^. We then have the following proposition.

**Proposition 12**

One has,

(60) $$\begin{array}{c} {𝒮}_{P}=A{𝒮}_{Q}A^{-1}. \end{array}$$

**Proof**

We have ${𝒮}_{P}=P\bar{P^{-1}}=P𝕊P^{-1}$, where *𝕊* is the shift matrix with ones on the super-diagonal and zeros elsewhere, with a similar expression for *𝒮*_*Q*_. We then have

(61) 𝒮P=P𝕊P−1=AQ𝕊(AQ)−1=AQ𝕊Q−1A−1=A𝒮QA−1.

## 4. The Chebyshev-Boubaker Connection Coefficients

We let

(62) $$\begin{array}{c} P=\left(\frac{1+ux+vx^{2}}{1+ax+bx^{2}},\frac{x}{1+ax+bx^{2}}\right),\\ Q=\left(\frac{1+sx+tx^{2}}{1+cx+dx^{2}},\frac{x}{1+cx+dx^{2}}\right). \end{array}$$

For the matrix of connection coefficients **A**, we recall that we have **A** = **P** **Q** ^−1^. Now, **P** and **Q** ^−1^ have the following factorizations:

(63) P=(1+ux+vx2,x)·(11+bx2,x1+bx2) ·(11+ax,x1+ax),Q−1=(11−cx,x1−cx)·(c(dx2),xc(dx2)) ·(11+sx+tx2,x).

We now proceed to list the elements of the factors involved. The matrix (1/(1 + *ax*), *x*/(1 + *ax*)) is the generalized inverse binomial matrix with general element

(64) $$\begin{array}{c} \left(\frac{1}{1+ax},\frac{x}{1+ax}\right)={\left(\left(\begin{array}{c} n\\ k \end{array}\right){\left(-a\right)}^{n-k}\right)}_{0\leq n,k\leq \infty }. \end{array}$$

The matrix (1/(1 + *bx* ^2^), *x*/(1 + *bx* ^2^)) gives the coefficients of the Chebyshev polynomials $U_{n}(\sqrt{b}x)$. We have

(65) (11+bx2,x1+bx2)  =((n+k2k)(−b)(n−k)/21+(−1)n−k2)0≤n,k≤∞.

The matrix (1 + *ux* + *vx* ^2^, *x*) is the banded matrix with the elements (1, *u*, *v*) occupying the three diagonals from the main diagonal down (with zeros elsewhere). The elements of the matrix product (1 + *ux* + *vx* ^2^, *x*) · (1/(1 + *bx* ^2^), *x*/(1 + *bx* ^2^)) are then given by

(66) (n+k2k)(−b)(n−k)/21+(−1)n−k2  +u(n+k−12k)(−b)(n−k−1)/21−(−1)n−k2  +v(n+k−22n−k−22)(−b)(n−k−2)/21+(−1)n−k2.

Let us denote these elements by *T*_*n*,*k*_. Then, the matrix **P** has elements given by

(67) $$\begin{array}{c} p_{n,k}=\sum_{j=0}^{n}T_{n,j}\left(\begin{array}{c} j-k\\ k \end{array}\right){\left(-a\right)}^{j-k}. \end{array}$$

A similar analysis of the factors in the expression for **Q** ^−1^ shows that the general element of **Q** ^−1^ is given by

(68) qn,k∗=∑i=0n ‍∑j=0n(nj)cn−ji+1j+1(j+1j−i2)    ×d(j−i)/21+(−1)j−i2∑r=0⌊(i−k)/2⌋(i−k−rr)    ×(−s)i−k−r(−t)r,

since we have

(69) (11−cx,x1−cx)=((nk)cn−k)0≤n,k≤∞,(c(dx2),xc(dx2))  =(k+1n+1(n+1n−k2)d(n−k)/21+(−1)n−k2)0≤n,k≤∞,(11+sx+tx2,x)  =(∑j=0⌊(n−k)/2⌋(n−k−jj)(−s)n−k−j(−t)j)0≤n,k≤∞.

We gather our results in the following proposition.

**Proposition 13**

Let

(70) $$\begin{array}{c} P=\left(\frac{1+ux+vx^{2}}{1+ax+bx^{2}},\frac{x}{1+ax+bx^{2}}\right),\\ Q=\left(\frac{1+sx+tx^{2}}{1+cx+dx^{2}},\frac{x}{1+cx+dx^{2}}\right) \end{array}$$

be the coefficient arrays of two families of Chebyshev-Boubaker polynomials. Then, the connection coefficients *a*_*n*,*k*_ for *P*_*n*_ in terms of *Q*_*n*_ are given by

(71) $$\begin{array}{c} a_{n,k}=\sum_{j=0}^{n}p_{n,j}q_{j,k}^{*}, \end{array}$$

where

(72) $$\begin{array}{c} p_{n,k}=\sum_{j=0}^{n}T_{n,j}\left(\begin{array}{c} j-k\\ k \end{array}\right){\left(-a\right)}^{j-k}, \end{array}$$

with *T*_*n*,*k*_ given by the expression

(73) (n+k2k)(−b)(n−k)/21+(−1)n−k2  +u(n+k−12k)(−b)(n−k−1)/21−(−1)n−k2  +v(n+k−22n−k−22)(−b)(n−k−2)/21+(−1)n−k2,qn,k∗=∑i=0n ‍∑j=0n(nj)cn−ji+1j+1(j+1j−i2)    ×d(j−i)/21+(−1)j−i2∑r=0⌊(i−k)/2⌋(i−k−rr)    ×(−s)i−k−r(−t)r.

The connection coefficient elements in the Chebyshev-Boubaker class of polynomials are given by Riordan arrays. The elements of these arrays can be expressed in terms of recurrences defined by the associated production matrices. Thus, we are naturally interested in the structure of *𝒮*_**A**_, the production matrix of **A**.

**Proposition 14**

Let **A** be the matrix of connection coefficients that express the polynomials *P*_*n*_(*x*) with coefficient array **P** in terms of the polynomials *Q*_*n*_(*x*) with coefficient array **Q**. Then, one has

(74) $$\begin{array}{c} {𝒮}_{A}=Q{𝒮}_{P}Q^{-1}. \end{array}$$

**Proof**

We have

(75) 𝒮A=A−1𝕊A=(PQ−1)−1𝕊(PQ−1)=QP−1𝕊PQ−1=Q𝒮PQ−1.

Proposition 7 can now be interpreted in this context as follows, giving a set of recurrence relations for the connection coefficients.

**Proposition 15**

If the *A* and *Z* sequences of *𝒮*_**A**_ are denoted, respectively, by *α*_*n*_ and *z*_*n*_, then the connection coefficients *a*_*n*,*k*_ satisfy the following recurrences

(76) $$\begin{array}{c} a_{n+1,k+1}=\sum_{j=0}^{n-k}\alpha_{j}a_{n,k+j},\\ a_{n+1,0}=\sum_{j=0}^{n}z_{j}a_{n,j}. \end{array}$$

## 5. Examples

In this section, we give examples of the foregoing, using simple examples that are close to the Chebyshev polynomials of the second kind. This enables us to illustrate the theory without an unnecessarily high overhead.

**Example 16**

We take the example of the families of orthogonal polynomials that have the aerated Catalan and the ordinary Catalan numbers as moments, respectively. Thus, let **P** = **S**, the coefficient array of the monic Chebyshev polynomials of the second kind. We have

(77) $$\begin{array}{c} S=\left(\frac{1}{1+x^{2}},\frac{x}{1+x^{2}}\right). \end{array}$$

We will have

(78) $$\begin{array}{c} Q=\left(\frac{1}{1+x},\frac{x}{{\left(1+x\right)}^{2}}\right). \end{array}$$

This is the array of the Morgan-Voyce polynomials $B_{n}(x)=\sum_{k=0}^{n}\left(\begin{array}{c} n+k\\ 2k \end{array}\right){\left(-1\right)}^{n-k}x^{k}$. Then,

(79) A=SQ−1=(11+x2,x1+x2)·(11+x,x(1+x)2)−1=(11+x2,x1+x2)·(c(x),xc(x)2).

This implies that

(80) $$\begin{array}{c} A=\left(\frac{1}{1+x^{2}}c\left(\frac{x}{1+x^{2}}\right),\frac{x}{1+x^{2}}c{\left(\frac{x}{1+x^{2}}\right)}^{2}\right). \end{array}$$

The matrix **A** begins with

(81) $$\begin{array}{c} \left(\begin{array}{ccccccc} 1 & 0 & 0 & 0 & 0 & 0 & \cdots \\ 1 & 1 & 0 & 0 & 0 & 0 & \cdots \\ 1 & 3 & 1 & 0 & 0 & 0 & \cdots \\ 3 & 7 & 5 & 1 & 0 & 0 & \cdots \\ 9 & 19 & 17 & 7 & 1 & 0 & \cdots \\ 25 & 57 & 55 & 31 & 9 & 1 & \cdots \\ \vdots & \vdots & \vdots & \vdots & \vdots & \vdots & \ddots \end{array}\right). \end{array}$$

The first column terms *a*_*n*_ = *a*_*n*,0_ have generating function

(82) $$\begin{array}{c} \frac{1}{1+x^{2}}c\left(\frac{x}{1+x^{2}}\right)=\frac{1+x^{2}-\sqrt{1-4x+2x^{2}-4x^{3}+x^{4}}}{2x\left(1+x^{2}\right)}. \end{array}$$

This form of g.f. suggests that this sequence may have an interesting Hankel transform. In fact, the sequence *a*_*n*,0_ is A101499, a sequence which gives the number of peakless Motzkin paths of length *n* in which the (1,0)-steps at levels greater than level 0 come in two colors (Emeric Deutsch). The Hankel transform of *a*_*n*,0_ starts with

(83) 1,0,−4,−16,−64,0,4096,65536,1048576,0,−1073741824,….

This is A162547, which is a Somos-4 variant [27, 39] in the sense that we have

(84) $$\begin{array}{c} a_{n}=\frac{\left(4a_{n-1}a_{n-3}-4a_{n-2}^{2}\right)}{a_{n-4}},\;\left(n\neq 4k+1\right). \end{array}$$

We have

(85) $$\begin{array}{c} a_{n,k}=\sum_{j=0}^{n-k}\;‍\sum_{i=0}^{\left\lfloor j/2\right\rfloor }{\left(-1\right)}^{i}\left(\begin{array}{c} j-i\\ i \end{array}\right)C_{j-2i}\left(\begin{array}{c} n-j-1\\ n-k-j \end{array}\right)2^{n-k-j}. \end{array}$$

We note that due to the combinatorial interpretation of *a*_*n*,0_ and the positivity of the Catalan numbers, we can conclude that all the connection coefficients *a*_*n*,*k*_ are positive in this case.

We now find expressions for the elements *α*_*n*_ and *z*_*n*_ of the production matrix *𝒮*_**A**_ of **A**. 

We use the values

(86) g(x)=11+x2c(x1+x2)=1+x2−1−4x+2x2−4x3+x42x(1+x2),f(x)=x1+x2c(x1+x2)2=(1−x)2−1−4x+2x2−4x3+x42x,

along with (46) to find that

(87) $$\begin{array}{c} Z\left(x\right)=\frac{\sqrt{1-4x+2x^{2}-4x^{3}+x^{4}}+x^{2}-1}{2x},\\ A\left(x\right)=\frac{{\left(1+x\right)}^{2}+\sqrt{1-4x+2x^{2}-4x^{3}+x^{4}}}{2}. \end{array}$$

This implies that

(88) zn=(1n)−∑k=0⌊(n−1)/2⌋1k+1(n−k−2k) ×(n−k−1k)(−2)n−1−2k,αn=(2n)−∑k=0⌊(n−2)/2⌋1k+1(n−k−3k) ×(n−k−2k)(−2)n−2−2k.

With these values for *z*_*n*_ and *α*_*n*_, we thus obtain the following recurrences for the connection coefficients:

(89) $$\begin{array}{c} a_{n+1,k+1}=\sum_{j=0}^{n-k}\alpha_{j}a_{n,k+j},\;\left(k,n=0,1,\ldots \right),\\ a_{n+1,0}=\sum_{j=0}^{n}z_{j}a_{n,j},\;\left(n=0,1,\ldots \right). \end{array}$$

We note that, in this case, the sequence elements *α*_*n*_ and *z*_*n*_ are essentially diagonal sums of generalized Narayana triangles [40].

**Example 17**

In this example, we let *P*_*n*_ be the family of orthogonal polynomials with the Catalan numbers *C*_*n*_ as moments, and we let *Q*_*n*_ be the family of orthogonal polynomials with the central binomial coefficients $\left(\begin{array}{c} 2n\\ n \end{array}\right)$ A000984 as moments. We find that

(90) $$\begin{array}{c} P=\left(\frac{1}{1+x^{2}},\frac{x}{1+x^{2}}\right),\\ Q=\left(\frac{1-x}{1+x^{2}},\frac{x}{1+x^{2}}\right). \end{array}$$

We obtain

(91) $$\begin{array}{c} A=PQ^{-1}=\left(\frac{1}{1-x},x\right), \end{array}$$

which is the partial sum matrix (the lower-triangular matrix all of whose nonzero elements are 1). This corresponds to

(92) $$\begin{array}{c} P_{n}\left(x\right)=\sum_{k=0}^{n}Q_{k}\left(x\right). \end{array}$$

In fact, we have

(93) $$\begin{array}{c} Q_{n}\left(x\right)=\left(\begin{array}{c} n+k\\ n-k \end{array}\right){\left(-1\right)}^{n-k}-\left(\begin{array}{c} n+k-1\\ n-k-1 \end{array}\right){\left(-1\right)}^{n-k-1},\\ P_{n}\left(x\right)=\left(\begin{array}{c} n+k\\ n-k \end{array}\right){\left(-1\right)}^{n-k}=\left(\begin{array}{c} n+k\\ 2k \end{array}\right){\left(-1\right)}^{n-k}=\sum_{k=0}^{n}Q_{k}\left(x\right). \end{array}$$

**Example 18**

Let *P*_*n*_(*x*) = *S*_*n*_(*x*), the monic Chebyshev polynomials of the second kind with the aerated Catalan numbers as moments, and let *Q*_*n*_(*x*) be the family of orthogonal polynomials with $\left(\begin{array}{c} 2n\\ n \end{array}\right)$ as moments. We get

(94) A=(11+x2,x1+x2)·(1−x1+x,x(1+x)2)−1=(11+x2,x1+x2)·(11−4x,xc(x)2)=(11+x211−4(x/(1+x2)),x1+x2c(x1+x2)2)=(11−4x+2x2−4x3+x4,  (1−x)2−1−4x+2x2−4x3+x42x).

The sequence *a*_*n*_ = *a*_*n*,0_ is then A101500, with

(95) $$\begin{array}{c} a_{n}=\sum_{k=0}^{\left\lfloor n/2\right\rfloor }\left(\begin{array}{c} n-k\\ k \end{array}\right)\left(\begin{array}{c} 2\left(n-2k\right)\\ n-2k \end{array}\right){\left(-1\right)}^{k}. \end{array}$$

In this example, we have

(96) $$\begin{array}{c} Q^{-1}={\left(\frac{1-x}{1+x},\frac{x}{{\left(1+x\right)}^{2}}\right)}^{-1}=\left(\left(\begin{array}{c} 2n\\ n-k \end{array}\right)\right), \end{array}$$

which is A094527. Hence, we have

(97) $$\begin{array}{c} a_{n,k}=\sum_{j=0}^{n}\left(\begin{array}{c} \frac{n+j}{2}\\ j \end{array}\right){\left(-1\right)}^{\left(n-j\right)/2}\frac{1+{\left(-1\right)}^{n-j}}{2}\left(\begin{array}{c} 2j\\ j-k \end{array}\right). \end{array}$$

Note that this implies that

(98) $$\begin{array}{c} a_{n}=\sum_{j=0}^{n}\left(\begin{array}{c} \frac{n+j}{2}\\ j \end{array}\right){\left(-1\right)}^{\left(n-j\right)/2}\frac{1+{\left(-1\right)}^{n-j}}{2}\left(\begin{array}{c} 2j\\ j \end{array}\right). \end{array}$$

**Example 19**

In this final example, we let **P** be the coefficient array of the Boubaker polynomials. Thus,

(99) $$\begin{array}{c} P=\left(\frac{1+3x^{2}}{1+x^{2}},\frac{x}{1+x^{2}}\right). \end{array}$$

We recall that the Boubaker polynomials can be expressed as

(100) $$\begin{array}{c} P_{n}\left(x\right)=\sum_{k=0}^{\left\lfloor n/2\right\rfloor }\left(\begin{array}{c} n-k\\ k \end{array}\right)\frac{n-4k}{n-k}{\left(-1\right)}^{k}x^{n-2k}. \end{array}$$

We let **Q** be the coefficient array of the Morgan-Voyce polynomials

(101) $$\begin{array}{c} Q_{n}\left(x\right)=\sum_{k=0}^{n}\left(\begin{array}{c} n+k\\ 2k \end{array}\right){\left(-1\right)}^{n-k}x^{k}. \end{array}$$

Then,

(102) $$\begin{array}{c} Q=\left(\frac{1}{1+x},\frac{x}{{\left(1+x\right)}^{2}}\right). \end{array}$$

The connection coefficients of the Boubaker polynomials in terms of the Morgan-Voyce polynomials are the elements of the array

(103) $$\begin{array}{c} A=\left(\frac{1+3x^{2}}{1+x^{2}},\frac{x}{1+x^{2}}\right)\cdot {\left(\frac{1}{1+x},\frac{x}{{\left(1+x\right)}^{2}}\right)}^{-1}. \end{array}$$

Thus,

(104) A=((1+3x2)(1+x2−1−4x+2x2−4x3+x4)2x(1+x2),  (1−x)2−1−4x+2x2−4x3+x42x).

Letting

(105) $$\begin{array}{c} T_{n,k}=\sum_{j=0}^{n-k}\;‍\sum_{i=0}^{\left\lfloor j/2\right\rfloor }{\left(-1\right)}^{i}\left(\begin{array}{c} j-i\\ i \end{array}\right)C_{j-2i}\left(\begin{array}{c} n-j-1\\ n-k-j \end{array}\right)2^{n-k-j}, \end{array}$$

(see (85)), we obtain

(106) $$\begin{array}{c} a_{n,k}=T_{n,k}+3T_{n-2,k}. \end{array}$$

Again, we see that all the coefficients *a*_*n*,*k*_ are positive.

## 6. Conclusions

We have shown that in the case of orthogonal polynomials defined by Riordan arrays, we can give both closed form expressions and recurrence relations for the connection coefficients, using the machinery from the theory of Riordan arrays.
